# Antimycin A Improves Heat Tolerance in Creeping Bentgrass by Suppressing Nighttime Respiration and Reprogramming Carbon and Nitrogen Metabolism

**DOI:** 10.1111/ppl.71151

**Published:** 2026-10-20

**Authors:** Amir Abbas, Stephanie Rossi, Bingru Huang

**Affiliations:** ^1^ Department of Plant Biology Rutgers University New Brunswick New Jersey USA

**Keywords:** amino acids, antimycin a, creeping bentgrass, heat stress, metabolomics, respiration

## Abstract

High temperature causes an increase in plant respiration, which may contribute to growth decline under heat stress. The objectives of this study were to investigate whether suppressing heat‐elevated dark respiration with a respiration inhibitor, antimycin A (AA), could improve heat tolerance and alter carbon and nitrogen‐containing metabolite pools in creeping bentgrass ( *Agrostis stolonifera* “Penncross”). Plants received water or foliar AA under 25°C/15°C or 35°C/35°C day/night conditions for 35 days. Turf quality, photosynthesis (Pn), and nighttime respiration (Rn) were measured weekly, and leaf metabolites were analyzed at Day 35. Under 35°C/35°C, AA‐treated plants maintained higher turf quality from Days 14–35, higher Pn, and lower Rn than plants not treated with AA. AA treatment increased the relative levels of glucose, cellobiose, and multiple amino acids, including proline, glutamine, asparagine, and γ‐aminobutyric acid. Pyruvate and shikimate increased, whereas α‐ketoglutarate decreased; malate and isocitrate did not differ significantly. By combining physiological measurements with metabolite profiling, this study identifies carbohydrate conservation and changes in carbon and nitrogen metabolism as potential contributors to AA‐associated heat tolerance in creeping bentgrass.

## 1 Introduction

Heat stress inhibits plant growth by affecting physiological and metabolic processes, including photosynthesis and respiration (Wahid et al. 2007). High temperatures can decrease photosynthetic carbon assimilation while increasing carbon consumption during respiration, although the respiratory response depends on the duration of heat stress and the capacity for thermal acclimation (Atkin and Tjoelker 2003). In creeping bentgrass, this imbalance between photosynthesis and respiration can contribute to carbohydrate depletion and a decline in turf quality during summer (Xu and Huang 2001; Huang et al. 2014). The decline mostly depends on the exposure duration of turfgrass to heat stress, its respiratory acclimation, and capacity to protect photosynthesis (Atkin and Tjoelker 2003; Huang et al. 2014). Understanding how changes in respiration are associated with carbohydrate retention and other metabolic responses is therefore important for identifying physiological processes that contribute to heat tolerance in this cool‐season turfgrass.

Respiration provides energy and metabolic intermediates required for plant growth and maintenance, but it also consumes carbohydrates and releases carbon as CO_2_ (Amthor 2000; Millar et al. 2011). Respiratory carbon consumption continues during the night, when photosynthetic carbon fixation is absent, making nighttime respiration an important component of plant carbon balance (Gifford 2003). In closely mown turfgrass, particularly on golf greens, carbohydrate depletion is more concerning because elevated temperatures and frequent mowing can limit the plant's capacity to meet energy demands, resulting in turf quality decline (Huang et al. 2014). These relationships provide a rationale for examining whether limiting heat‐associated respiratory carbon loss, while maintaining daytime photosynthesis, could help preserve carbohydrates and improve heat tolerance. Because respiration also supports essential cellular functions, the physiological consequences of its inhibition must be considered together with changes in plant growth and metabolism.

Antimycin A (AA) is a naturally occurring depsipeptide antibiotic produced by bacteria, primarily from the genus *Streptomyces* (Joynt and Seipke 2018). It is a mitochondrial respiration inhibitor that restricts electron flow through Complex III of the cytochrome respiratory chain (Quinlan et al. 2011). Plant mitochondria also contain alternative oxidase (AOX), which accepts electrons from ubiquinol and transfers them to oxygen, bypassing Complexes III and IV. This alternative pathway allows electron transport to continue when the cytochrome pathway is restricted, but it conserves less energy through proton translocation (Millar et al. 2011). However, the contribution of this pathway depends on its regulation and electron partitioning, so an enhanced AOX activity cannot be inferred solely from exposure to AA (Vanlerberghe 2013).

The effects of AA on plant stress responses have been studied under multiple abiotic stresses, including heat, cold, flooding, and salinity (Batjuka 2019; Batjuka et al. 2014, 2017; Batjuka and Skute 2017, 2021; Bui et al. 2020; Erdal et al. 2015). A study on wheat ( *Triticum aestivum* L.) reported effects of AA on pigment composition and the functional activity of the photosynthetic apparatus (Batjuka et al. 2017). AA also inhibits an AA‐sensitive component of cyclic electron transport around photosystem I. Consequently, its effects on intact plants can involve both mitochondria and chloroplasts (Taira et al. 2013). Another study on wheat ( *Triticum aestivum* L.) has reported greater leaf and coleoptile length, higher fresh and dry weight, and enhanced relative water content compared with control plants following AA treatment during heat stress (Batjuka and Skute 2021). These findings provide a physiological rationale for testing AA, but how the physiological response is associated with changes in leaf carbon and nitrogen metabolism in creeping bentgrass remains unresolved.

Metabolite profiling helps to understand how physiological responses are linked to changes in carbon and nitrogen metabolism. Carbohydrates supply substrates for respiration, while organic acids play a key role in the tricarboxylic acid (TCA) cycle, connecting carbon oxidation with the provision of carbon skeletons for the synthesis of amino acids (Millar et al. 2011). Changes in mitochondrial function can therefore be associated with the changes in both carbohydrate and amino acid pools, as evaluated in cucumber ( *Cucumis sativus* ) plants with altered mitochondrial genomes (Szal et al. 2010). γ‐aminobutyric acid (GABA) application in creeping bentgrass was found to increase heat tolerance and was linked with changes in sugars, amino acids, and organic acids, together with an improved osmotic adjustment and antioxidant metabolism (Li et al. 2016). Similarly, the foliar application of proline or GABA on creeping bentgrass improved turf quality during heat stress and altered amino acid profiles (Rossi et al. 2021). These findings support examining metabolites such as carbohydrates, organic acids, and amino acids together when investigating the responses linked with heat tolerance.

Although previous studies provide evidence for beneficial physiological responses to AA, they do not establish how AA‐associated changes in CO_2_ release relate to leaf carbon and nitrogen metabolic profiles when creeping bentgrass is subjected to prolonged heat stress. The question addressed here was whether improved heat tolerance following foliar application of AA would be accompanied by conservation of carbohydrates and changes in organic acid and amino acid pools. AA was therefore used as an experimental tool under controlled conditions to examine relationships among canopy CO_2_ exchange, leaf metabolites, and heat tolerance.

Creeping bentgrass ( *Agrostis stolonifera* ) was selected because it is an economically important cool‐season perennial grass used on golf course putting greens, where sensitivity to high temperature creates challenges for maintaining summer turf quality (Huang et al. 2014). Its persistent canopy permits repeated assessments of treatment responses during prolonged heat stress. We hypothesized that suppressing cellular respiration with AA could improve heat tolerance by reducing the carbon loss and enhancing the accumulation of carbohydrates and other carbon‐related compounds. The main goal of this study was to determine whether suppressing respiration with the foliar application of AA could improve heat tolerance in creeping bentgrass ( *Agrostis stolonifera* ), a cool‐season perennial grass species, in association with reduced nighttime CO_2_ release. A second objective was to identify associated changes in leaf carbohydrates, organic acids and amino acids. Together, these measurements were used to examine the relationship between canopy CO_2_ exchange, leaf metabolite levels and heat tolerance under controlled conditions.

## 2 Materials and Methods

### 2.1 Plant Materials and Growth Conditions

Sods of creeping bentgrass (cv. “Penncross”) were collected from Horticulture Research Farm II, North Brunswick, NJ, USA. Sods were transplanted in 16 pots (5 cm in diameter and 22 cm in height). Plants were established for 28 days in the greenhouse under natural sunlight supplemented with sodium vapor lamps, with temperatures maintained at 24°C/16°C (day/night). Canopies were trimmed by hand once weekly to 4 cm. Pots were watered every other day to field capacity and supplied with three‐quarter‐strength Hoagland's nutrient solution every 14 days (Hoagland and Arnon 1950).

Plants were then acclimated for 7 days in a growth chamber set to 25°C/15°C (day/night), 60% relative humidity, and a photosynthetic photon flux density of 620 μmol m^−2^ s^−1^ at the canopy level. The photoperiod was 12 h, with light from 05:00 to 17:00 and darkness from 17:00 to 05:00. During the 35‐day experiment, plants were maintained in growth chambers at either 25°C/15°C or 35°C/35°C day/night. Vapor‐pressure deficit was not monitored during the experiment. The specific CO_2_ concentrations and lamp spectral information were unavailable.

### 2.2 Experimental Design and AA Application

This experiment used a split‐plot treatment arrangement, with two temperature treatments as the main‐plot factor and two chemical treatments as the subplot factor with four replicates (pots of plants) for each treatment. Each temperature regime contained four water‐treated control pots and four AA‐treated pots. Pots were randomized and repositioned within their assigned chamber. Each regime occupied one chamber in a single experimental run. Antimycin A (Sigma‐Aldrich, A8674) was prepared fresh before each application by dissolving 4.389 mg in approximately 100 μL of 95% ethanol and adding it to 200 mL distilled water, giving a nominal 40 μM concentration and the final ethanol concentration of 0.05% (v/v).

The AA treatment, designated 40 μM, was selected through an unpublished preliminary screening experiment that compared multiple concentrations before this experiment. The selected concentration produced the most favorable treatment response in that screening. Previous research has also used 40 μM AA in *Arabidopsis thaliana* leaf infiltration experiments investigating chloroplast ATP synthase regulation (Buchert et al. 2021). AA was applied as 15 sprays per pot on the day before heat treatment and subsequently once weekly during the 35‐day study. Control plants were sprayed with water. The same application schedule was used under both heat and control temperature regimes.

### 2.3 Physiological Measurements

Turf quality (TQ), canopy photosynthesis (Pn), and nighttime respiration (Rn) were measured at Days 7, 14, 21, 28, and 35 on the same replicate pots during the experimental period. TQ was rated visually on a scale of 1–9, based on the color, uniformity, and density of the turf canopy, with 9 being the highest TQ (Morris and Shearman 1998). Canopy photosynthetic rate (Pn) and nighttime respiration rate (Rn) were measured every 7 days during the experimental period using a CO_2_ gas analyzer (Model EGM‐5, PP Systems), with an attached glass chamber (Model CPY‐5). The CO_2_ efflux was calculated based on the linear increase or decrease in CO_2_ concentration over 60 s.

Measurements were taken weekly on Thursdays between 10:00 and 15:00 during the daytime and between 18:00 and 22:00 at night. Nighttime measurements began 1 h after lights were switched off, and plants remained in darkness throughout the measurements.

### 2.4 Metabolomic Extraction and Liquid Chromatography–Mass Spectrometry (LC–MS) Analysis

Leaf tissue was collected from both untreated control and AA‐treated plants in both temperature regimes at Day 35 of heat stress, frozen in liquid nitrogen, and stored at −80°C in a freezer. Each biological sample represented one pot, with four samples per treatment and per regime. For further analysis, samples were freeze‐dried for 48 h and ground using a mortar and pestle. Samples were analyzed by the Rutgers metabolomics laboratory using the LC–MS methods described in Errickson and Huang (2023). Ground dry tissue, 20 mg per sample, was extracted in 1 mL of a 40:40:20 acetonitrile:methanol:water solution and left at ambient temperature for a period of 10 min. Subsequently, 50 μL of 15% (m/v) NH₄HCO₃ was added, and extracts were diluted four times with the extraction solvent. Samples were centrifuged at 16,000 × g for 10 min at 4°C, and the supernatants were collected for analysis in 5 mL labeled microcentrifuge tubes.

Chromatographic separation was performed using a Vanquish Horizon UHPLC system (Thermo Fisher Scientific) with an XBridge Amide column, 150 mm × 2.1 mm, 2.5 μm particle size (Waters) using two solvents. Solvent A contained 95%:5% H_2_O:acetonitrile with 20 mM acetic acid and 40 mM ammonium hydroxide (pH 9.4), while solvent B contained 20%:80% H_2_O:acetonitrile with 20 mM acetic acid and 40 mM ammonium hydroxide (pH 9.4). The gradient, expressed as time in minutes and percentage B, was 0 min, 100%; 3.2 min, 90%; 6.2 min, 90%; 6.5 min, 80%; 10.5 min, 80%; 10.7 min, 70%; 13.5 min, 70%; 13.7 min, 45%; 16 min, 45%; 16.5 min, 100%; and 22 min, 100%. Flow rate was 300 μL·min^−1^, column temperature at 25°C, autosampler temperature at 4°C, and an injection volume of 5 μL. Mass spectra were acquired in positive and negative ion modes at a resolution of 70,000 at *m/z* 200, with an automatic gain control target of 3 × 10^6^, and an *m/z* scan range of 72–1000. Data processing used MAVEN with a mass‐accuracy window of 5 ppm (Melamud et al. 2010).

### 2.5 Metabolite Normalization

Metabolite abundances were normalized using nine labeled internal standards: thymine‐^2^H_4_, inosine‐^15^N_4_, glucose‐^2^H_7_‐^13^C_6_, alanine‐^2^H_4_‐^15^N, glycine‐^13^C_2_‐^15^N, glutamine‐^2^H_5_‐^15^N_2_, serine‐^13^C_3_‐^15^N, malate‐^2^H_3_ and lysine‐^2^H_8_. Each standard's signal was divided by its mean across the analytical batch. These ratios were averaged within each sample, and metabolite signals were divided by the resulting normalization factor. Dry tissue input was standardized to 20 mg per sample, with identical extraction and dilution procedures. Laboratory‐normalized signals were analyzed without additional dry‐weight correction and are reported as relative metabolite abundances.

### 2.6 Statistical Analysis

The effect of AA and heat stress on TQ, canopy Pn, Rn, and metabolite content was performed with ANOVA, and treatments found to have significant differences were tested by Fisher's protected LSD post hoc test at a significance level of *α* = 0.05 in Python (version 3.12). The significant effect of AA on each metabolite was tested by ANOVA at a significance level of *α* = 0.05 in Python.

Laboratory‐normalized metabolite levels were analyzed at day 35 using the same two‐way model, without additional transformation. Cross‐regime comparisons assumed a common analytical scale. Benjamini–Hochberg adjustment was applied across the 36 metabolite measurements separately for each model term and each within‐temperature regime, AA versus control, with adjusted *p* < 0.05 considered significant. Metabolite bar graphs show means ± SE relative to the corresponding control mean; statistical tests used laboratory‐normalized relative metabolite levels.

Heatmaps were generated using log_2_‐transformed relative metabolite abundance, standardized as z scores for each metabolite within each temperature regime. Partial least squares discriminant analysis (PLS‐DA) was performed separately for each metabolite class and temperature regime using two components after log_2_‐transformation, mean centering, and unit‐variance scaling.

## 3 Results

### 3.1 Effects of Antimycin A on Turf Quality, Photosynthesis, and Respiration in Creeping Bentgrass

Under non‐stress conditions, TQ was stable regardless of treatment, and there were no significant differences between untreated and AA‐treated plants (Figure 1A). There was a decline overall in TQ in all plants subjected to heat stress; however, plants treated with AA maintained significantly higher TQ than that of untreated control plants from 14 to 35 days (Figure 1B).

**FIGURE 1:**
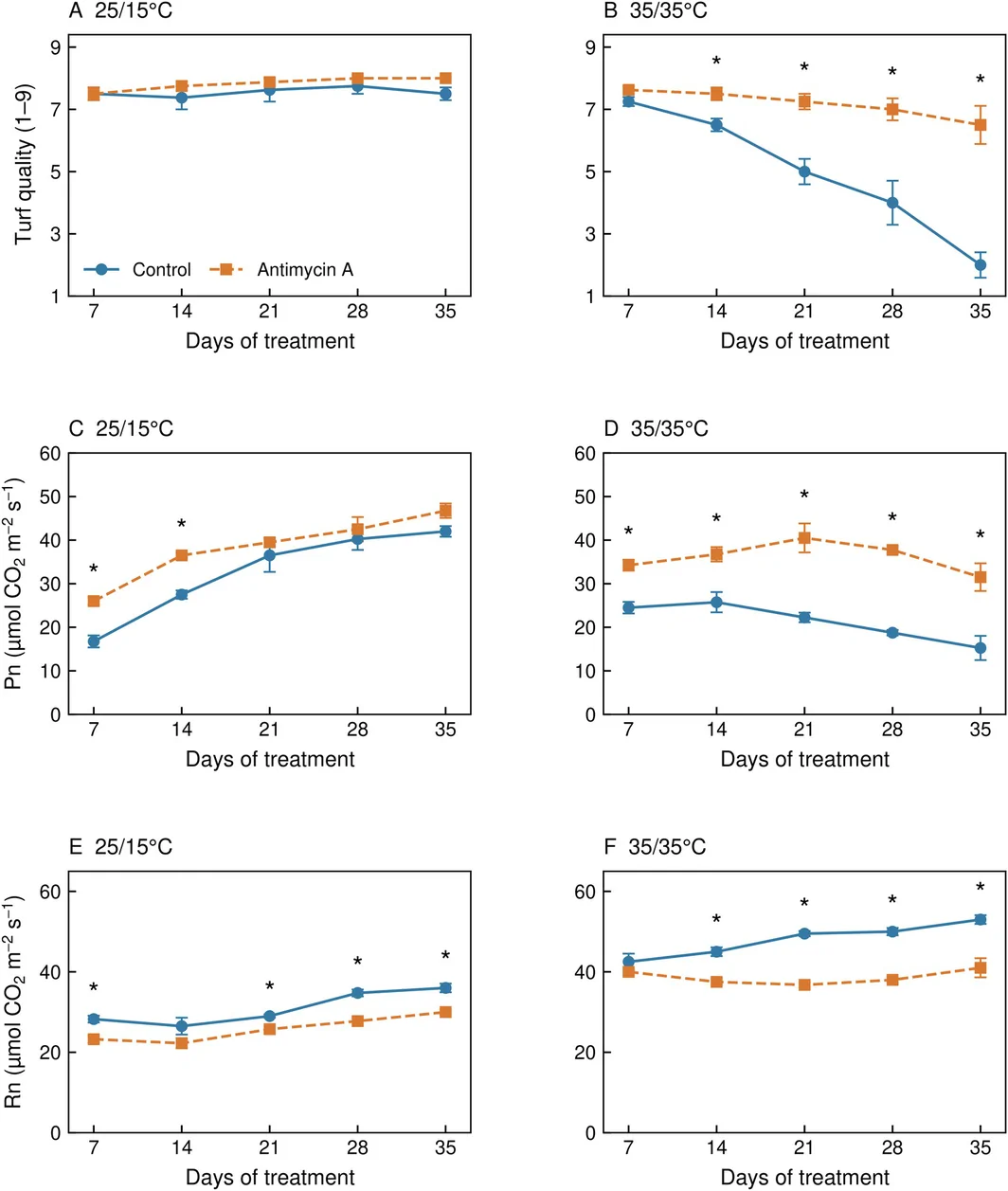
Effects of antimycin A (AA) on turf quality (A, B), photosynthesis (Pn; C, D) and nighttime respiration (Rn; E, F) in creeping bentgrass under 25°C/15°C (A, C, E) and 35°C/35°C (B, D, F) day/night temperatures. Values are means ± SE of four replicate pots. Asterisks indicate significant differences between AA and control at the same sampling date within each temperature regime (*p* < 0.05, treatment comparisons based on two‐way ANOVA).

Canopy Pn showed a rising trend in untreated and AA‐treated plants under non‐stress conditions and was significantly greater in AA‐treated plants at 7 and 14 days (Figure 1C). Under heat stress, the canopy Pn decreased in untreated control plants, whereas the AA‐treated plants were able to maintain a significantly higher Pn throughout the experiment (Figure 1D).

Plants receiving foliar application of AA maintained significantly lower canopy Rn than untreated controls at 7, 21, 28, and 35 days under non‐stress conditions (Figure 1E). The effects of AA on inhibiting dark respiration were more pronounced under heat stress than under non‐stress conditions. Although canopy Rn increased as heat stress proceeded, AA‐treated plants exhibited a significant reduction in this parameter compared with control plants from 14 to 35 days of heat stress (Figure 1F).

### 3.2 Carbohydrates and Related Compounds

Foliar application of AA significantly increased the relative levels of cellobiose, glucose 6‐phosphate, and glycerol 3‐phosphate compared to untreated plants under non‐stress conditions, while the remaining carbohydrates and related compounds showed no significant differences between treatments (Figure 3). Under heat stress, AA‐treated plants maintained significantly higher levels of glucose, cellobiose, glucose 6‐phosphate, UDP‐N‐acetylglucosamine, and UDP‐glucose as compared to control plants. The additional measurement of UDP‐N‐acetylglucosamine (NEG) was also found to be significantly higher in AA‐treated plants, whereas glucose 6‐phosphate (NEG) and glycerol 3‐phosphate did not differ significantly between treatments. NEG denotes an additional negative‐ion measurement (Figure 2).

**FIGURE 2:**
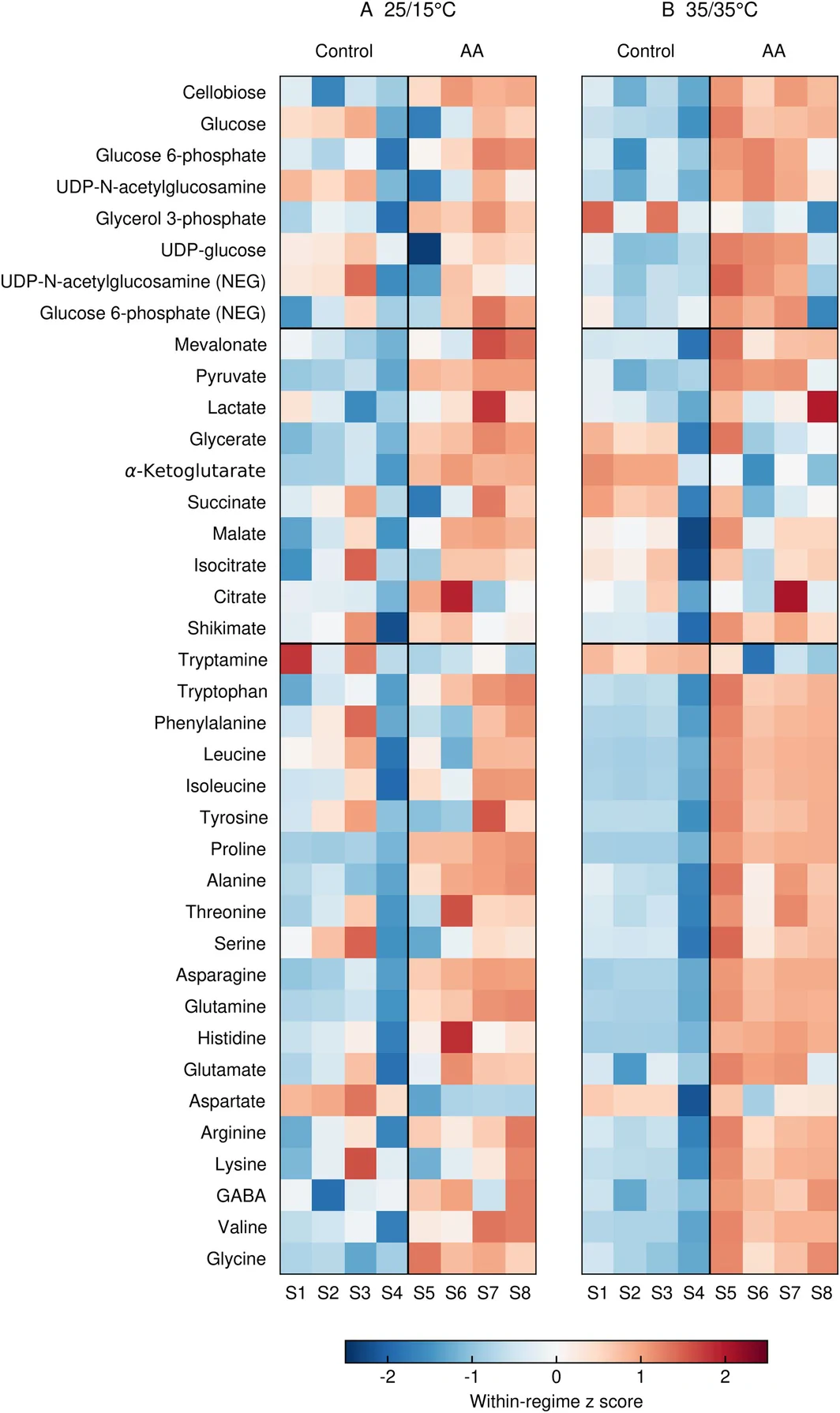
Heatmaps of relative metabolite abundances in creeping bentgrass leaves after 35 days under 25°C/15°C (A) and 35°C/35°C (B) day/night temperatures. S1–S4 represent control pots and S5–S8 represent antimycin A (AA)‐treated pots. Colors show *z* scores of log_2_‐transformed abundances calculated separately for each metabolite within each temperature regime; red and blue indicate higher and lower abundances, respectively. GABA, γ‐aminobutyric acid; NEG, additional negative‐ion measurement; UDP, uridine diphosphate.

### 3.3 Organic Acids

Under non‐stress conditions, foliar application of AA significantly increased the relative levels of pyruvate, glycerate, and α‐ketoglutarate as compared to control plants, while the remaining organic acids showed no significant differences between treatments (Figure 4). Under heat stress, AA‐treated plants showed significantly higher levels of mevalonate, pyruvate, lactate, and shikimate, and significantly lower levels of α‐ketoglutarate as compared to control plants. Malate, isocitrate, citrate, succinate, and glycerate did not show any significant differences between treatments.

### 3.4 Amino Acids and Tryptamine

Under non‐stress conditions, AA‐treated plants showed significantly higher levels of alanine and glycine and lower levels of aspartate as compared to control plants (Figure 5). The remaining amino acids and tryptamine showed no significant differences. Under heat stress, AA‐treated plants showed significantly higher levels of proline, valine, phenylalanine, glutamate, asparagine, isoleucine, leucine, glutamine, alanine, tryptophan, γ‐aminobutyric acid (GABA), histidine, threonine, tyrosine, serine, arginine, lysine, and glycine as compared to control plants (Figure 5). Aspartate did not differ significantly between treatments, whereas tryptamine showed a significantly lower level in AA‐treated plants.

### 3.5 PLS‐Da

The PLS‐DA score plots showed separation between untreated and AA‐treated plants for all three metabolite classes, carbohydrates, amino acids/organic acids, and tryptamine, under both stress and non‐stress conditions (Figure 6). These patterns indicated differences in metabolic profiles between treatments.

## 4 Discussion

### 4.1 Carbon Conservation Through Reduced Night Respiration

Antimycin A has been reported to improve plant tolerance to various abiotic stresses, including heat stress (Batjuka 2019; Batjuka et al. 2014, 2017; Batjuka and Skute 2017, 2021; Bui et al. 2020; Erdal et al. 2015). In our study, AA‐treated plants showed improved heat tolerance, which was linked with lower canopy Rn, higher canopy Pn, and higher TQ compared to control plants (Figure 1). We also recorded significantly higher levels of glucose, UDP‐glucose, and cellobiose in AA‐treated plants during heat stress compared to control plants (Figure 3). These physiological and metabolic responses suggest that reduced respiratory carbon loss, together with maintained photosynthesis, contributes to the preservation of carbohydrates during prolonged heat stress.

**FIGURE 3:**
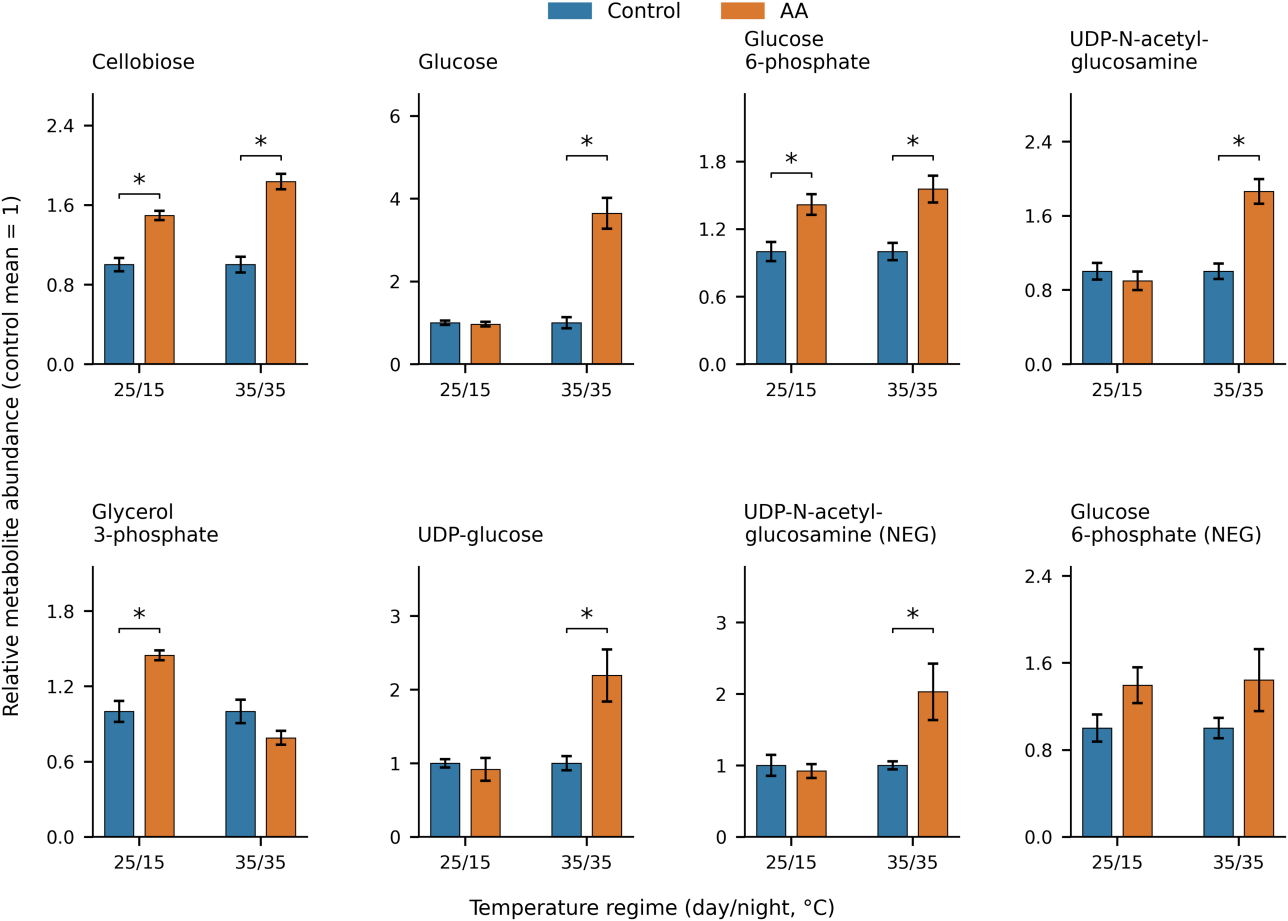
Effects of antimycin A (AA) on the relative abundances of carbohydrates and related compounds in creeping bentgrass leaves after 35 days under 25°C/15°C and 35°C/35°C day/night temperatures. Bars represent means ± standard error (SE) of four replicate pots, expressed relative to the control mean within each temperature regime (control = 1). Asterisks indicate significant differences between AA and control within each regime (two‐way ANOVA contrasts, Benjamini–Hochberg‐adjusted *p* < 0.05). NEG, additional negative‐ion measurement; UDP, uridine diphosphate.

Antimycin A inhibits mitochondrial Complex III at the ubiquinone reduction site, restricting electron transfer through the cytochrome respiratory pathway (Quinlan et al. 2011). A reduction in mitochondrial respiration could decrease carbohydrate oxidation and it might contribute to the accumulation of carbohydrates (Millar et al. 2011). However, this inhibition may also promote over‐reduction of electron carriers and reactive oxygen species (ROS) formation (Quinlan et al. 2011). The alternative oxidase (AOX) pathway transfers electrons from ubiquinol to oxygen, bypassing Complexes III and IV. Although less efficient for adenosine triphosphate (ATP) production, this pathway can limit over‐reduction and accumulation of ROS (Maxwell et al. 1999; Vanlerberghe 2013). These properties provide a possible biochemical explanation for the maintenance of cellular function when cytochrome respiration is restricted. It was also evaluated that AA influences the photosynthetic electron transport. Previous studies in isolated spinach ( *Spinacia oleracea* ) chloroplasts reported effects on photosynthetic induction and CO_2_ assimilation (Schacter et al. 1971; Miginiac‐Maslow and Champigny 1974), and the AA‐sensitive cyclic electron transport around photosystem I is another site of action (Taira et al. 2013). Therefore, it is plausible that the maintained canopy Pn in our study may reflect both direct effects on chloroplast processes as well as the preservation of leaf function under heat stress. The combined responses of both Pn and Rn suggest that AA limited respiratory carbon consumption while maintaining daytime photosynthetic activity.

Carbohydrate availability is associated with heat tolerance in creeping bentgrass (Li et al. 2022). Glucose is an important energy source, a signaling molecule involved in growth and stress responses, and a building block for polysaccharides, while UDP‐glucose supplies activated glucose for sucrose and synthesis of cell wall polysaccharides and may participate in signaling (Janse van Rensburg and Van den Ende 2018). The higher levels of these compounds, together with glucose 6‐phosphate in our study, could be the reason for continued metabolism and the maintenance of cellular structure during heat stress. Cellobiose, a disaccharide derived from cellulose breakdown, also links cell wall turnover with stress signaling. A study in lettuce ( *Lactuca sativa* ) reported enhanced defense responses following cellobiose treatment (He et al. 2023). In our study, the higher levels of cellobiose recorded under both temperature regimes suggest that AA affected carbohydrate metabolism even in the absence of heat stress. Taken together, all these responses support a contribution of carbohydrate conservation to improved heat tolerance.

### 4.2 Organic Acid Metabolism and Connections Between Carbon and Nitrogen Metabolism

Organic acids link respiratory metabolism to the supply of carbon skeletons for biosynthesis, and the response of these functions differs with the changes in mitochondrial activity (Szal et al. 2010; Tcherkez et al. 2009). In our study, AA‐treated plants had significantly higher levels of pyruvate, lactate, mevalonate, and shikimate, but lower α‐ketoglutarate during heat stress conditions (Figure 4). Under non‐stress conditions, both pyruvate and α‐ketoglutarate increased in AA‐treated plants. Thus, pyruvate increased under both temperature regimes, while α‐ketoglutarate responded differently under both temperature regimes.

**FIGURE 4:**
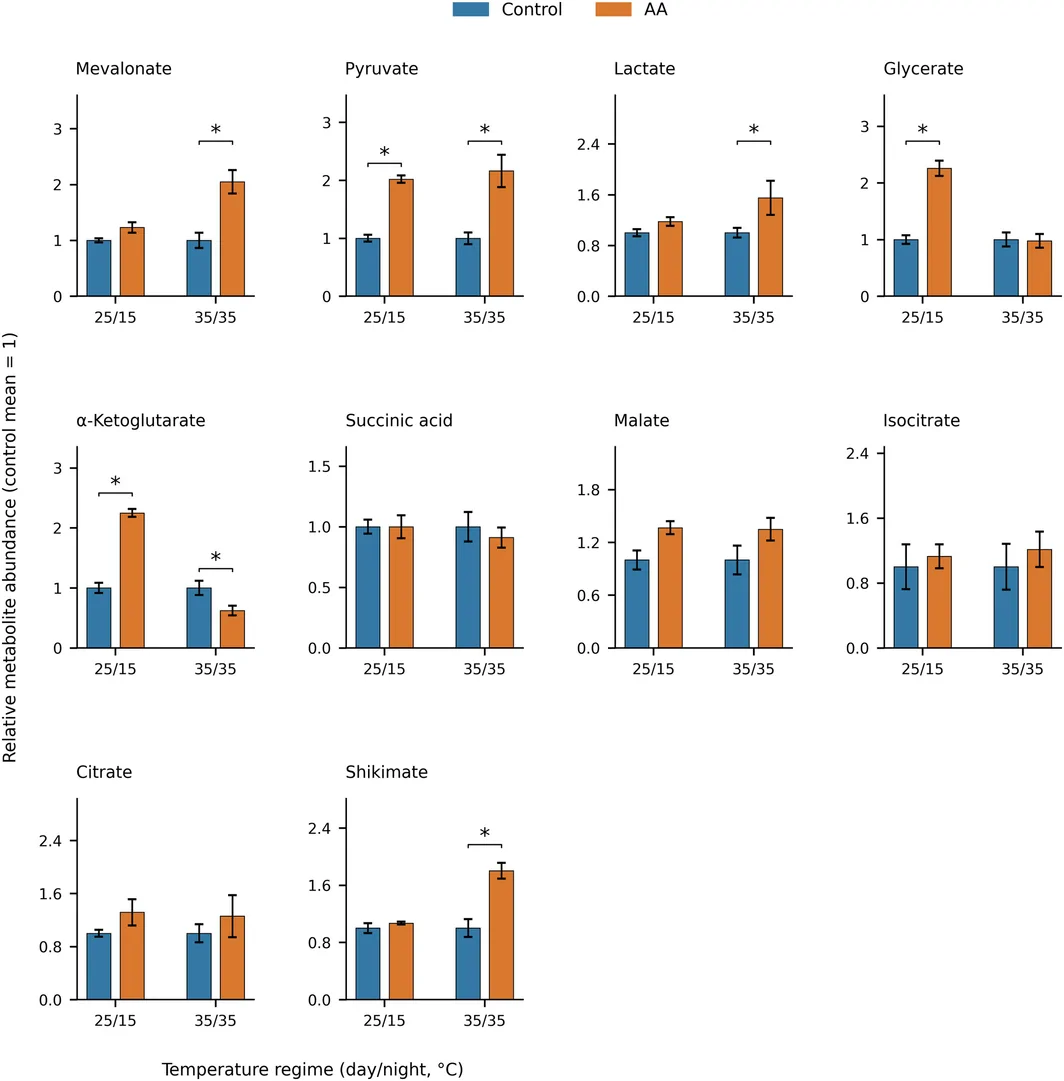
Effects of antimycin A (AA) on the relative abundances of organic acids in creeping bentgrass leaves after 35 days under 25°C/15°C and 35°C/35°C day/night temperatures. Bars represent means ± SE of four replicate pots, expressed relative to the control mean within each temperature regime (control = 1). Asterisks indicate significant differences between AA and control within each regime (two‐way ANOVA contrasts, Benjamini–Hochberg‐adjusted *p* < 0.05).

Pyruvate plays a key role in linking glycolysis with the mitochondrial carbon oxidation via the pyruvate dehydrogenase reaction, which supplies acetyl‐CoA to the TCA cycle (Szal et al. 2010). Reduction of pyruvate to lactate provides a route for reoxidizing NADH to NAD^+^ when the oxidation of mitochondria is restricted (Steinbeck et al. 2020). A study conducted in *Arabidopsis thaliana* , using a fluorescent NADH/NAD^+^ biosensor, found that the inhibition of mitochondrial electron transport by AA increased the reduction state of the cytosolic NAD pool (Steinbeck et al. 2020). In our study, the simultaneous accumulation of both lactate and pyruvate during heat stress (Figure 4) is therefore compatible with an adjustment in pyruvate utilization linked to respiratory and redox constraints (Steinbeck et al. 2020). This is a proposed explanation rather than a measured redox response.

The decreased level of α‐ketoglutarate provides a further connection between respiration and amino acid metabolism. Besides its role in the TCA cycle, α‐ketoglutarate provides the carbon skeleton for the formation of glutamate via the glutamine synthetase/glutamate synthase pathway (Szal et al. 2010). Isotope‐labeling experiments have found that the TCA pathway can operate in separate branches in illuminated leaves, supporting the production of biosynthetic intermediates rather than functioning exclusively as a complete oxidative cycle (Tcherkez et al. 2009). We recorded lower α‐ketoglutarate along with increased levels of glutamate, glutamine, proline, and GABA under heat stress (Figures 4 and 5). This pattern suggests a shift in the balance between respiratory carbon metabolism and the availability of nitrogen‐containing compounds, consistent with the connections established in previous experimental studies (Szal et al. 2010; Tcherkez et al. 2009).

**FIGURE 5:**
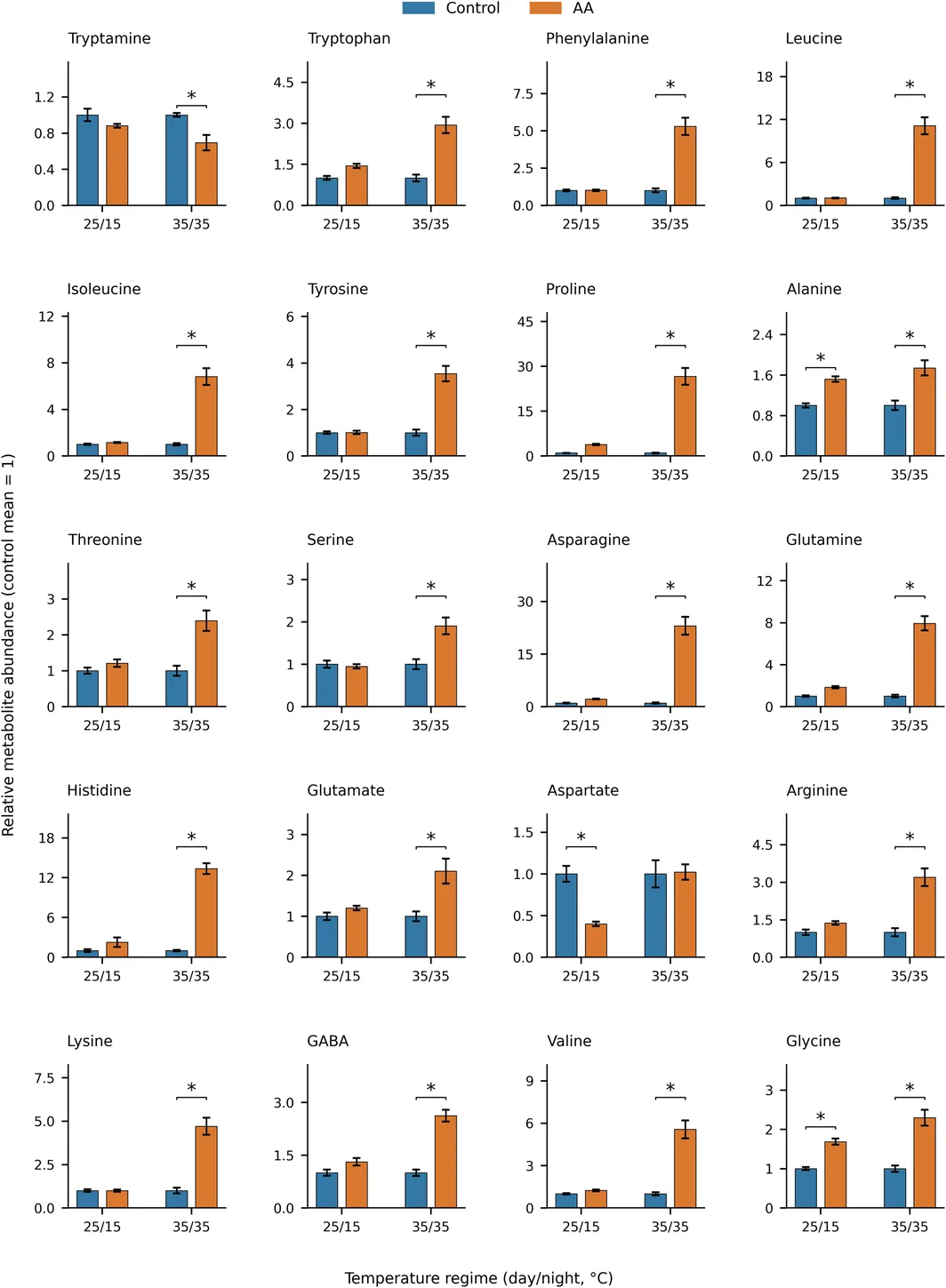
Effects of antimycin A (AA) on the relative abundances of amino acids and tryptamine in creeping bentgrass leaves after 35 days under 25°C/15°C and 35°C/35°C day/night temperatures. Bars represent means ± SE of four replicate pots, expressed relative to the control mean within each temperature regime (control = 1). Asterisks indicate significant differences between AA and control within each regime (two‐way ANOVA contrasts, Benjamini–Hochberg‐adjusted *p* < 0.05). GABA, γ‐aminobutyric acid.

**FIGURE 6:**
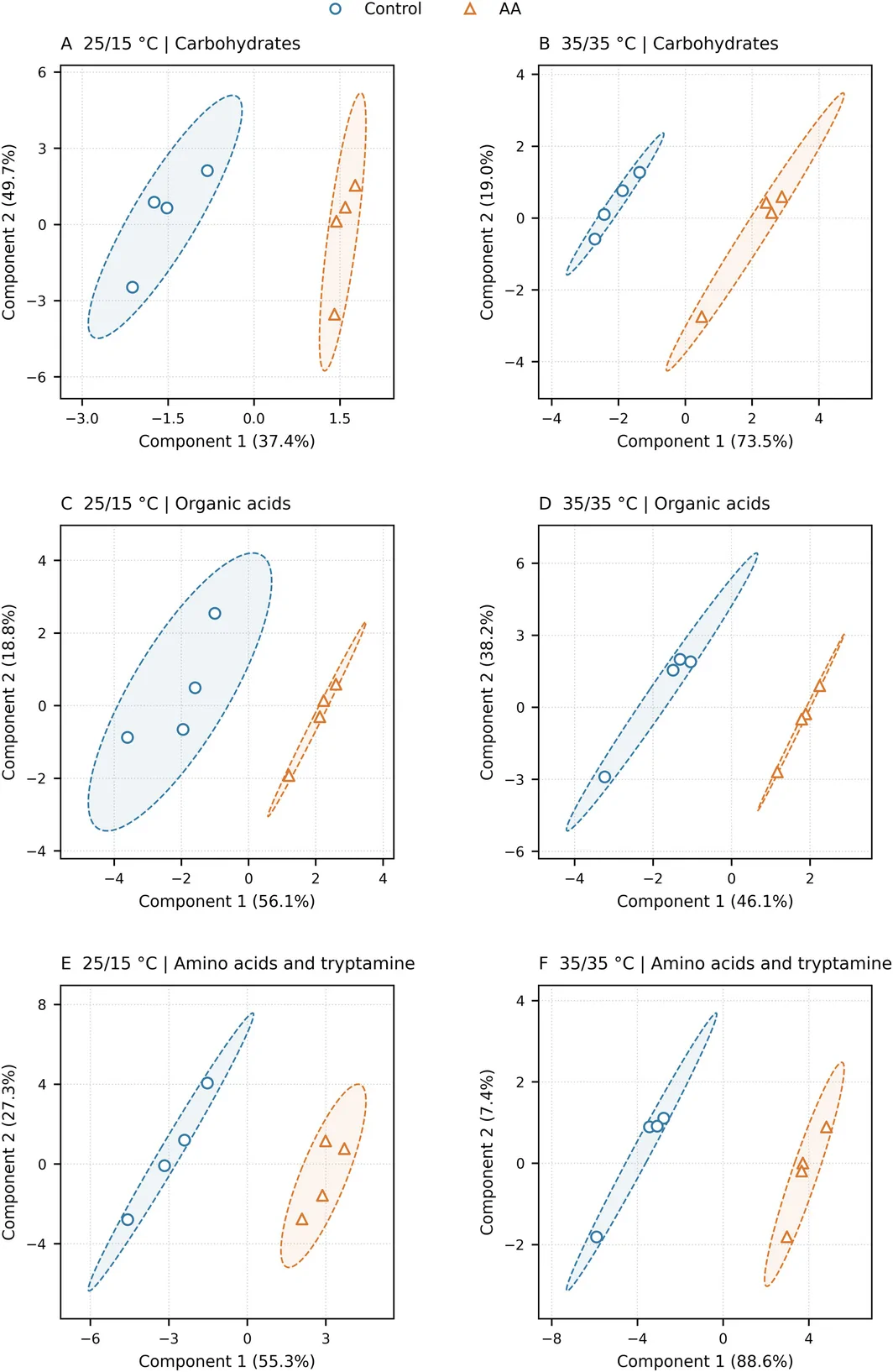
Partial least squares discriminant analysis (PLS‐DA) of carbohydrates (A, B), organic acids (C, D), and amino acids and tryptamine (E, F) in control and antimycin A (AA)‐treated creeping bentgrass after 35 days under 25°C/15°C (A, C, E) and 35°C/35°C (B, D, F) day/night temperatures. Each point represents one pot (*n* = 4 per treatment). Dashed ellipses show within‐group dispersion as 95% data ellipses. Axis percentages indicate the metabolite variance explained by each component.

**FIGURE 7:**
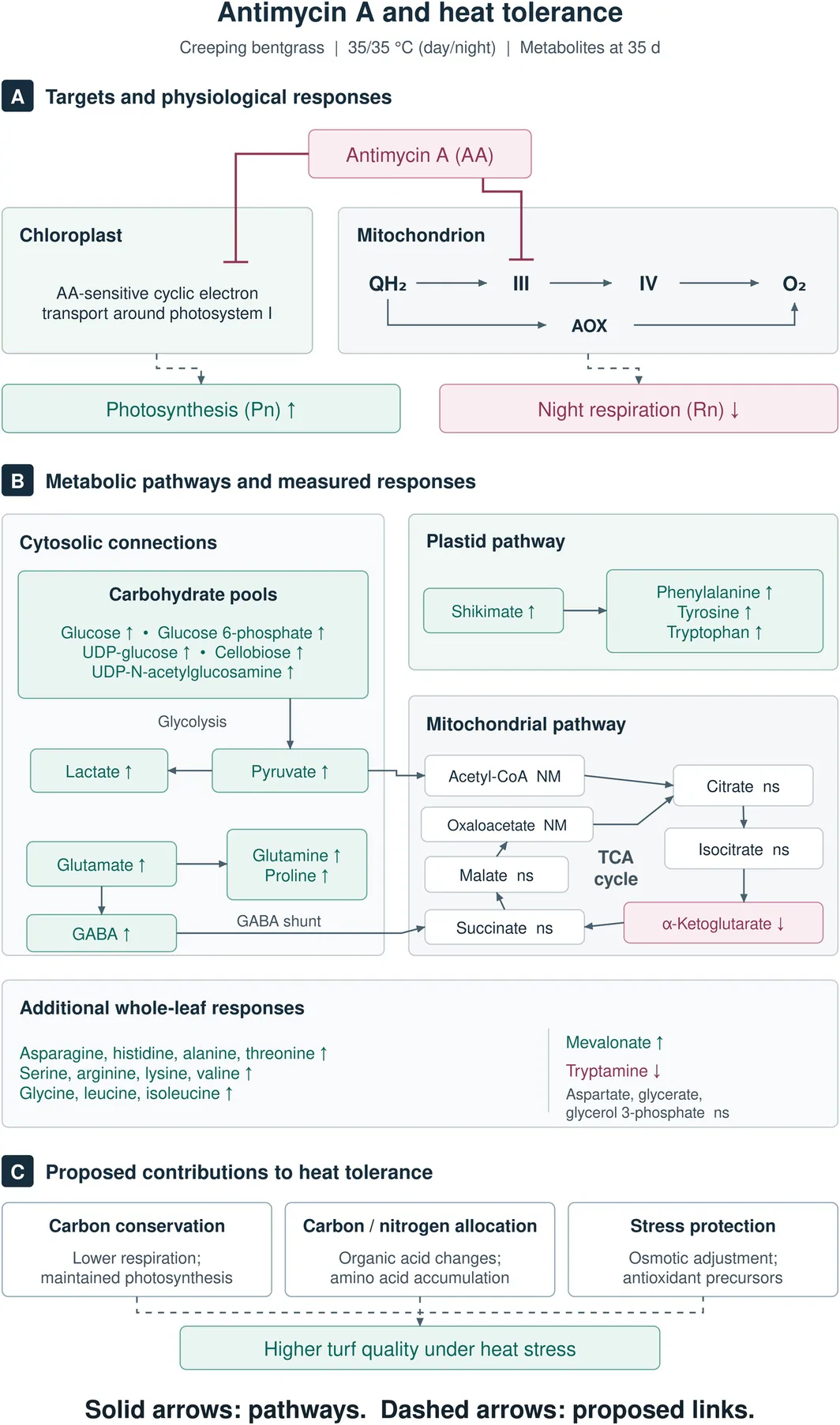
Proposed model of antimycin A (AA)‐associated heat tolerance in creeping bentgrass under 35°C/35°C day/night temperatures. Panels show known AA targets and observed physiological responses (A), metabolic pathways and whole‐leaf metabolite changes at Day 35 (B), and proposed contributions to heat tolerance (C). In panel B, upward and downward arrows indicate higher and lower abundances in AA‐treated plants relative to controls (Benjamini–Hochberg‐adjusted *p* < 0.05); ns, not significant; NM, not measured. Solid arrows indicate biochemical connections and dashed arrows indicate proposed links. Compartments provide pathway context for the whole‐leaf measurements. AOX, alternative oxidase; CoA, coenzyme A; GABA, γ‐aminobutyric acid; III and IV, mitochondrial electron‐transport Complexes III and IV; QH_2_, ubiquinol; TCA, tricarboxylic acid; UDP, uridine diphosphate.

The increased levels of mevalonate in our study show that the organic acid response also involved a precursor outside of the TCA cycle (Figure 4). Mevalonate is involved in the cytosolic isoprenoid pathway, which supplies the precursors for the biosynthesis of sterol; inhibitor experiments in *Arabidopsis thaliana* demonstrated the connections between this pathway and sterol accumulation (Laule et al. 2003). Higher accumulation of mevalonate in AA‐treated plants thus identifies a change in the availability of this isoprenoid precursor, whose biosynthetic role has been established experimentally (Laule et al. 2003).

The shikimate pathway connects primary metabolism with the biosynthesis of tyrosine, phenylalanine, and tryptophan, while the phenylalanine supplies the phenylpropanoid pathway (Oliva et al. 2021). A study on tobacco ( *Nicotiana tabacum* ) reported that increased levels of aromatic amino acids were associated with greater accumulation of phenylpropanoid metabolites (Oliva et al. 2021). In our study, we recorded higher levels of shikimate, phenylalanine, tyrosine, and tryptophan in AA‐treated plants as compared to control plants (Figures 4 and 5). The coordinated accumulation of shikimate and aromatic amino acids during heat stress suggests greater availability of precursors for phenylpropanoid biosynthesis, providing a basis for examining a possible contribution of this pathway to the AA response (Oliva et al. 2021).

### 4.3 Amino Acid Accumulation and Its Potential Contribution to Heat Tolerance

This study found that AA‐treated plants exhibited higher accumulation of most of the measured amino acids during heat stress, including proline, GABA, histidine, glutamine, asparagine, and the aromatic amino acids (Figure 5). Previous experimental studies in creeping bentgrass reported that the exogenous application of proline and GABA improved heat tolerance and altered amino acid metabolism, providing experimental support for examining these compounds in relation to the responses observed here (Li et al. 2016; Rossi et al. 2021).

The accumulation of proline has been experimentally reported to protect plants during osmotic stress and control of oxidative injury (Székely et al. 2008). A study in *Arabidopsis thaliana* reported that mutants impaired in stress‐induced proline biosynthesis were more sensitive to salt stress and accumulated more ROS (Székely et al. 2008). Another experimental study conducted in creeping bentgrass reported that proline application improved turf quality and chlorophyll retention during heat stress (Rossi et al. 2021). In our study, the higher accumulation of proline was linked with maintained Pn and higher TQ in AA‐treated plants (Figures 1 and 5). These responses support a possible contribution of proline in protection against stress, consistent with the evidence from proline‐treated creeping bentgrass (Rossi et al. 2021).

GABA provides a connection between amino acid and respiratory metabolism through the GABA shunt, which links the metabolism of glutamate to succinate formation (Li et al. 2016). Li et al. (2016) demonstrated that application of GABA improved the heat tolerance in creeping bentgrass, along with higher photosynthesis and osmotic adjustment, lower electrolyte leakage, and changes in the antioxidant system of ascorbate–glutathione. In our study, the concurrent increases in GABA and glutamate (Figure 5) suggest that AA affected metabolite pools in the TCA cycle (Figure 7). The increased levels of GABA in AA‐treated plants may contribute to heat tolerance via osmotic adjustment and antioxidant protection, consistent with responses reported following GABA application in creeping bentgrass (Li et al. 2016).

Glutamine and asparagine provide additional connections with nitrogen assimilation and redistribution (Gaufichon et al. 2013; Moison et al. 2018). Genetic and ^15^N‐labeling experiments in *Arabidopsis thaliana* demonstrated that cytosolic glutamine synthetase contributes to assimilation of ammonium and nitrogen remobilization (Moison et al. 2018). Gaufichon et al. (2013) also demonstrated that disruption of asparagine synthetase reduced asparagine in phloem sap and nitrogen transfer to sink tissues. We recorded higher levels of glutamine and asparagine in AA‐treated plants during heat stress (Figure 5), indicating larger pools of compounds involved in nitrogen assimilation and transport. Their accumulation could retain nitrogen in forms available for subsequent redistribution, consistent with the reduced asparagine in phloem sap and nitrogen transfer to sink tissues previously noted (Gaufichon et al. 2013; Moison et al. 2018).

Histidine accumulation was also linked with improved heat tolerance in our study (Figures 1 and 5). A similar association was reported in hard fescue, where the heat‐tolerant cultivar “Reliant IV” exhibited higher levels of histidine, glutamine, proline, threonine, aspartate, and tryptophan as compared to the heat‐sensitive cultivar “Predator” during heat stress (Wang et al. 2018). This comparison supports the association between the accumulation of histidine and heat tolerance observed in our study and previously reported by others (Wang et al. 2018). Together with the aromatic amino acid precursors discussed in Section 4.2, the accumulation of GABA, proline, glutamine, and asparagine provides a biochemical basis for considering stress protection and retention of nitrogen as possible contributors to the improved turf performance (Li et al. 2016; Gaufichon et al. 2013; Moison et al. 2018; Rossi et al. 2021).

Collectively, lower Rn, maintained Pn, and higher levels of carbohydrate occurred alongside changes in organic acid pools and accumulation of amino acids in AA‐treated plants (Figures 1, 3, 4, 5). These findings support a working model in which carbohydrate conservation is accompanied by adjustments in carbon and nitrogen metabolism and the availability of potentially protective metabolites, as summarized in Figure 7. The experimental evidence for GABA and proline‐mediated heat tolerance in creeping bentgrass provides a foundation for this interpretation, while the specific contribution of each pathway to the AA response remains to be established (Li et al. 2016; Rossi et al. 2021).

### 4.4 Study Limitations and Practical Implications

Metabolites were measured in whole‐leaf extracts at the end of the 35‐day study period. Their levels indicate changes in the metabolite pools but do not establish subcellular distribution, pathway flux, or rates of biosynthesis. This study did not measure AOX activity, ROS levels, phenolic compounds, antioxidant function, or daily carbon balance, so the proposed mechanisms require direct testing. The AA concentration used in this experiment was selected through preliminary screening of multiple concentrations, based on the most favorable treatment response. The study examined a selected concentration of AA in one cultivar, with repeated applications throughout the 35‐day study period. Persistence of the treatment effects after AA applications stopped was not evaluated. Field trials using AA have already been conducted over two consecutive years, and their unpublished results will be reported separately to assess treatment performance under field conditions.

## 5 Conclusion

Foliar application of AA improved heat tolerance in creeping bentgrass, as indicated by higher TQ, maintained Pn, and lower Rn during prolonged heat stress. These responses were accompanied by higher levels of carbohydrate, increased pyruvate and shikimate, decreased α‐ketoglutarate, and accumulation of amino acids, including proline, GABA, histidine, and aromatic amino acids. By combining canopy measurements with leaf metabolite profiling, this study links AA‐associated heat tolerance with coordinated changes in carbon and nitrogen metabolism. The findings of this study support conservation of carbohydrate and an increased availability of metabolites involved in stress protection as potential contributors to improve turf performance during heat stress conditions.

## Author Contributions

**Amir Abbas:** data curation, formal analysis, investigation, methodology, and writing original draft. **Stephanie Rossi:** writing, review and editing. **Bingru Huang:** conceptualization, funding acquisition, methodology, project administration, resources, supervision, and writing, review and editing.

## Funding

This work was supported by New Jersey Agricultural Experiment Station (NJAES) and Center for Turfgrass Science.

## Disclosure

No Generative AI was used. Grammarly was used to edit grammar and structure of sentences in the text.

## Conflicts of Interest

The authors declare no conflicts of interest.

## Supporting information


**Table S1:** Inventory of 36 metabolite measurements representing 36 compounds in creeping bentgrass leaves.

## Data Availability

The data that support the findings of this study are available on request from the corresponding author. The data are not publicly available due to privacy or ethical restrictions.
