# Supplementary material for: Antimycin A Improves Heat Tolerance in Creeping Bentgrass by Suppressing Nighttime Respiration and Reprogramming Carbon and Nitrogen Metabolism
Source: Physiol Plant. 2026 Oct 20;178(5):e71151. doi: 10.1111/ppl.71151 (PMC13682501; doi:10.1111/ppl.71151)
Supplement: Supplementary file 1 [file PPL-178-e71151-s001.pdf]

Supporting information

Antimycin A improves heat tolerance in creeping bentgrass by suppressing nighttime respiration and reprogramming carbon and nitrogen metabolism

Amir Abbas, Stephanie Rossi and Bingru Huang

Department of Plant Biology and Pathology, Rutgers University, New Brunswick, NJ, USA

Contents

Table S1. Mass spectrometry properties of metabolites.

No.	RT (min)	Metabolite	m/z
1	7.47	GABA	102.06
2	2.60	Adenine	134.05
3	2.72	Adenosine	266.09
4	6.63	Alanine	88.04
5	8.32	Alpha-ketoglutarate	145.01
6	7.81	Asparagine	131.05
7	9.05	Aspartate	132.03
8	11.33	Citrate	191.02
9	5.88	Glucose	179.06
10	11.78	Glucose 6-phosphate	259.02
11	10.81	Glucose 1-phosphate	259.02
12	8.48	Glutamate	146.05
13	8.90	Glutamine	145.06
14	6.03	Glycerate	105.02
15	7.11	Glycine	74.02
16	9.17	Histidine	154.06
17	9.95	Isocitrate	191.02
18	4.45	Isoleucine	130.09
19	4.42	Lactate	89.02
20	4.03	Leucine	130.09
21	11.04	Lysine	145.10
22	9.24	Malate	133.01
23	3.43	Mevalonate	147.07
24	3.69	Phenylalanine	114.06
25	5.77	Proline	114.06
26	2.82	Pyruvate	87.01
27	7.81	Serine	104.04
28	8.46	Shikimate	173.04
29	8.81	Succinic acid	117.02
30	6.94	Threonine	118.05
31	3.47	Tryptophan	203.08
32	5.17	Tyrosine	180.08
33	2.27	Tryptamine	161.1
34	5.47	Valine	116.07

RT, retention time; m/z, mass-to-charge ratio; GABA, γ-aminobutyric a
